# A critical assessment of the “sterile womb” and “in utero colonization” hypotheses: implications for research on the pioneer infant microbiome

**DOI:** 10.1186/s40168-017-0268-4

**Published:** 2017-04-28

**Authors:** Maria Elisa Perez-Muñoz, Marie-Claire Arrieta, Amanda E. Ramer-Tait, Jens Walter

**Affiliations:** 1 Department of Agriculture, Food and Nutritional Sciences, 4-126 Li Ka Shing Centre for Health Research Innovation, University of Alberta, Edmonton, Alberta T6G 2E1 Canada; 2 Department of Physiology and Pharmacology, University of Calgary, Cumming School of Medicine, 3330 Hospital Drive NW, Calgary, Alberta T2N 4N1 Canada; 3 Department of Pediatrics, University of Calgary, Cumming School of Medicine, 3330 Hospital Drive NW, Calgary, Alberta T2N 4N1 Canada; 4 Department of Food Science and Technology, 260 Food Innovation Center, University of Nebraska-Lincoln, 1901 N 21st Street, Lincoln, Nebraska 68588-6205 USA; 5 Department of Biological Sciences, 7-142 Katz Group Centre, University of Alberta, Edmonton, Alberta T6G 2E1 Canada

**Keywords:** Sterile womb, In utero colonization, Microbiome, Placenta, Contamination, Axenic animals

## Abstract

After more than a century of active research, the notion that the human fetal environment is sterile and that the neonate’s microbiome is acquired during and after birth was an accepted dogma. However, recent studies using molecular techniques suggest bacterial communities in the placenta, amniotic fluid, and meconium from healthy pregnancies. These findings have led many scientists to challenge the “sterile womb paradigm” and propose that microbiome acquisition instead begins in utero, an idea that would fundamentally change our understanding of gut microbiota acquisition and its role in human development. In this review, we provide a critical assessment of the evidence supporting these two opposing hypotheses, specifically as it relates to (i) anatomical, immunological, and physiological characteristics of the placenta and fetus; (ii) the research methods currently used to study microbial populations in the intrauterine environment; (iii) the fecal microbiome during the first days of life; and (iv) the generation of axenic animals and humans. Based on this analysis, we argue that the evidence in support of the “in utero colonization hypothesis” is extremely weak as it is founded almost entirely on studies that (i) used molecular approaches with an insufficient detection limit to study “low-biomass” microbial populations, (ii) lacked appropriate controls for contamination, and (iii) failed to provide evidence of bacterial viability. Most importantly, the ability to reliably derive axenic animals via cesarean sections strongly supports sterility of the fetal environment in mammals. We conclude that current scientific evidence does not support the existence of microbiomes within the healthy fetal milieu, which has implications for the development of clinical practices that prevent microbiome perturbations after birth and the establishment of future research priorities.

## Background

The gastrointestinal tract of humans is colonized by a dense microbial community that has co-evolved with its host to become a vital component of our biology. The host-microbiome interrelationship is therefore considered a mutualistic symbiosis, with the human body providing sustenance and an adequate physical environment for the microbial populations, while the microbes execute essential functions, such as aiding in immune system development and providing defense against enteric infections [1].

Research in both animal models and humans suggests that the process of microbial colonization is especially significant during early life, as this period constitutes a critical window for immunological and physiological development [2, 3]. Given the importance of microbial symbionts to their host’s development and survival, mechanisms must be in place to facilitate their reliable transmission [4]. Symbiont transmission has been well-established in many host-microbial symbioses, especially in invertebrates (i.e., insects, nematodes, and the Hawaiian squid *Euprymna scolopes*) where it ranges from being strictly vertical (maternal) to horizontal (transmission between members of the same species or the environment) [5]. In contrast to these models of symbiosis, the modes of transmission for the more complex microbiomes of humans and other vertebrates are more intricate and incompletely understood. Considering the importance of the pioneer infant microbiota for human development and biology, it is essential that we elucidate the exact mechanisms by which this community is acquired, the time-points when colonization events occur, and the endogenous and exogenous factors that influence these events.

The degree of sterility of the fetal environment and the possibility of in utero microbiome transfer have been debated for almost 150 years [6]. In the second half of the last century, the field reached a consensus that the fetus was maintained in a sterile state [7]. According to this concept, which has been referred to as the *sterile womb paradigm* [4], microbes are acquired both vertically (from the mother) and horizontally (from other humans or the environment) during and after birth. However, there is now a multitude of recent studies employing modern sequencing technologies that have challenged the traditional view of human microbiome acquisition. These studies propose that neither the fetus, the placenta, nor the amniotic fluid are sterile, and that acquisition and colonization of the human gastrointestinal tract begins in utero [8–10]. If this “in utero colonization hypothesis” proves correct, there would be major repercussions on our understanding of the establishment of the pioneer human microbiome, its role in human health and the role of environmental, lifestyle, and clinical factors that affect its assembly and function. This concept would also have significant implications on how we view the fundamental aspects of host-microbial symbiosis in humans as well as clinical practices such as cesarean sections (C-sections), which are currently thought to disrupt transmission of microbes [11].

In this review, we first describe the scientific evidence in support of both the “sterile womb” and in utero colonization hypotheses. We then compare and critically assess the two opposing ideas and discuss the limitations of the research supporting each of them. We especially put effort into the historic perspective on this topic, with equal focus on both the older literature and more recent studies. Based on this assessment, we conclude that most of the evidence is in support of the “sterile womb hypothesis,” and we discuss the implications for clinical practice and future research.

## The traditional view: the sterile womb paradigm

**Fig. 1:**
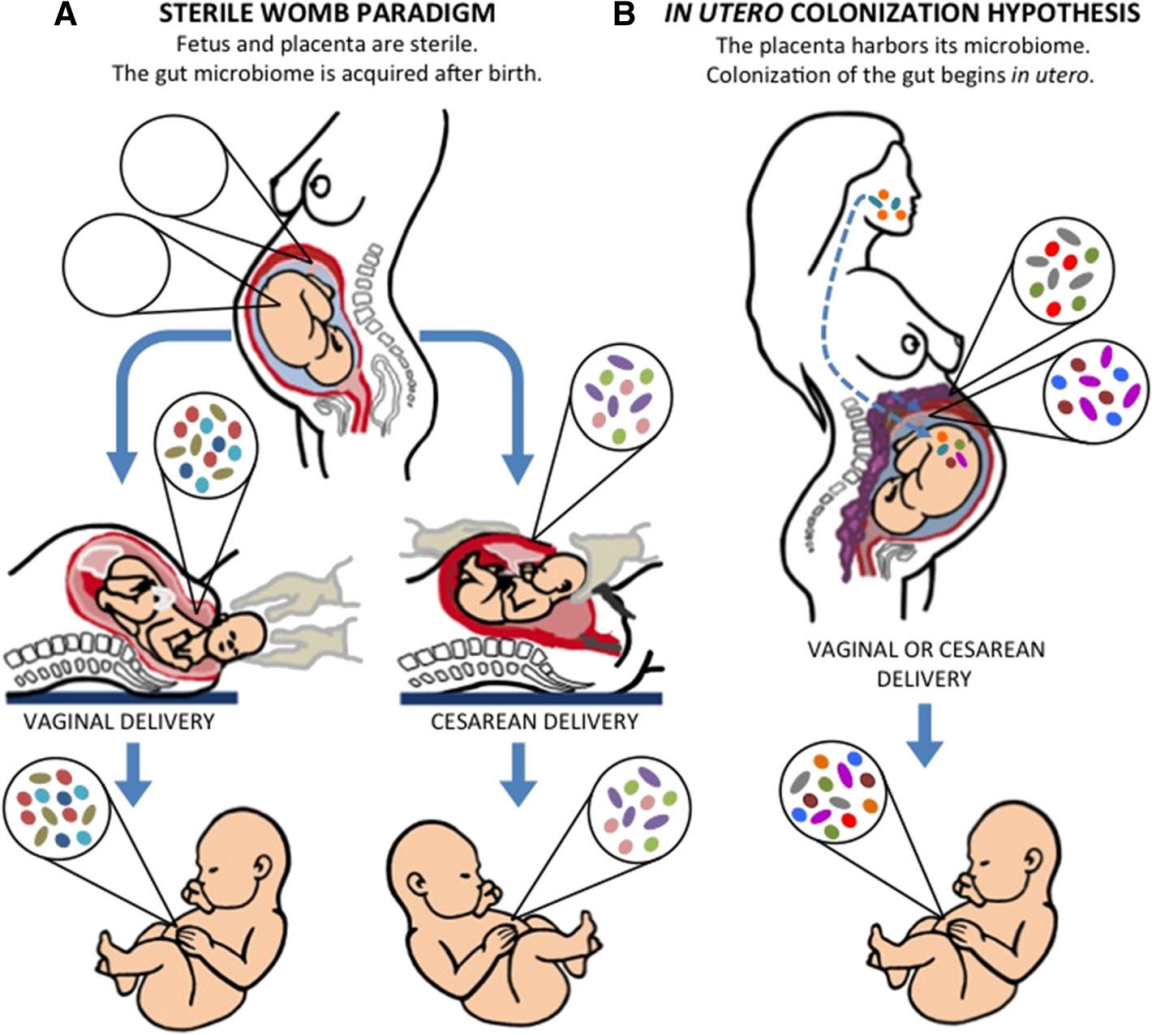
Schematic representation of the opposing concepts by which human microbiota is acquired early in life. **a** In the sterile womb paradigm, the placenta, amniotic fluid, and fetal gut remain sterile during a healthy pregnancy, and the early microbiome is acquired during and after birth. Accordingly, the gut microbiota of infants born vaginally resemble the microbiota of the mother’s vagina, while the microbiota of infants born by cesarean section are similar to the mother’s skin microbiota. **b** The “in utero colonization hypothesis” proposes that some microbial members of the infants’ gut microbiome are acquired before birth, probably via contact with a placental microbiome, which has been suggested to originate from the mother’s gut or oral microbiome

Most studies that established the sterile womb paradigm date back to research that employed traditional culture-based methods and microscopy, which despite their limitations are still considered valid today. As early as 1885, Theodor Escherich described the meconium (the earliest stool from an infant) to be free of viable bacteria [7], suggesting that the human fetus develops within a sterile environment (Fig. 1a). Later, two additional, independent studies conducted in 1927 and 1934 (*n* = 100 and *n* = 50, respectively) using sterile diapers for collection both found 62% of meconium samples from healthy pregnancies to be negative for bacteria by aerobic and anaerobic culture [12, 13]. The observed time frame for meconium expulsion ranged from a few minutes to 26 h after birth, with 50% of the meconiums expelled 5 to 10 h post birth [13]. Interestingly, the 1934 study reported a positive correlation between the detection of bacteria and time elapsed between birth and meconium passage [13]. Some recent studies utilizing molecular approaches also suggest that the majority of meconium samples from healthy pregnancies are negative for bacteria (Table 1). In one study [14], meconium samples from 66% (*n* = 15) of newborns evaluated showed evidence of bacteria based on fluorescence in situ hybridization (FISH), while only 7% were positive by PCR. Interestingly, four out of the five samples that were delivered within 500 min of birth showed no detectable bacteria by FISH, supporting the association between time of meconium passage and bacterial detection. Overall, these studies suggest that early meconium harbors no detectable bacteria, while later samples do, indicating the need to account for time elapsed post-birth when investigating bacterial presence in these samples.

**Table 1:** Summary of recent studies on the microbiology of amniotic fluid, placenta, and meconium

Sample type	Population	Methods	Significant findings and/or authors’ conclusions	Reference
Placental membranes, umbilical venous blood	Term and preterm vaginal and elective cesarean deliveries, (preterm deliveries include pregnancies complicated with preeclampsia, fetal growth restriction, or prolonged labor), deliveries that presented PPROM (*n* = 52)	FISH using generic probes for 16S rRNA genes	Bacteria were detected in 70% of placentas. Authors concluded presence of bacteria is common in placental membranes, but insufficient to cause preterm labor or PPROM	[37]
Meconium passed within the first 2 h of life	Term healthy newborns (*n* = 21)	Culture methods, Gram staining, 16S rDNA sequencing	Bacterial species isolated from one single meconium samples varied from 1 to 5. *Enterococcus faecalis* was the most abundant species found in 80% of the samples. Samples clustered by processing time	[8]
Placental membranes	Full-term and preterm vaginal and cesarean deliveries; preterm deliveries with and without PROM (*n* = 74)	Standard PCR of 16S rRNA gene and quantitative PCR for selected bacteria	Bacterial DNA was detected in 30% of placental tissue by standard PCR, while 43% were positive by qPCR; 14% were positive by both methods. No bacterial DNA was detected in C-section deliveries at term, while 50% of term vaginal deliveries were positive	[93]
Placenta	Full-term vaginal and cesarean deliveries from pregnant women participating in diet study (*n* = 34)	Aerobic and anaerobic cultures. PCR of 16 s rRNA gene using genus and species specific primers for *Lactobacillus* and bifidobacteria	DNA was detected in 94% of samples by PCR. Bacteria of interest were not detected by culture methods	[84]
Meconium and feces	Preterm neonates (*n* = 23)	454 pyrosequencing	Bacterial DNA was detected in 91% of samples. Lower gestational age was associated with lower bacterial diversity, but there are no differences in diversity between C-section and vaginally delivered infants	[98]
Amniotic fluid, placenta and meconium	Elective cesarean deliveries of healthy mothers enrolled in probiotic study (*n* = 43)	Quantitative PCR for selected bacterial groups (*Lactobacillus*, *Bifidobacterium*, *Bacteroides*, *Clostridium leptum* group)	Lactobacillus DNA was found in 100% of placentas, *Bifidobacterium* in 41%, *Bacteroides* in 34% and *Clostridium leptum* in 31%. DNA for the selected bacterial groups was found in 43% of amniotic fluid samples	[51]
Meconium passed between 2–48 h after birth	Infants born by vaginal or cesarean deliveries from diabetic and non-diabetic mothers (*n* = 23)	16S rRNA sequencing using Pacbio RS system	Bacteria were found in 100% of samples. Diversity is lower in meconium when compared to adults; higher in infants from diabetic when compared to non-diabetic mothers	[99]
Meconium	Healthy full-term deliveries (*n* = 20)	Pyrosequencing of 16S rRNA gene	Meconium microbiota differed from the microbiota of feces, vagina, and skin from adults but was similar to that of young infant feces. Meconium microbiota has an intrauterine origin and is influenced by maternal factors.	[149]
Maternal feces, meconium, baby’s feces at different timepoints	Healthy mothers, full-term pregnancies, all infants exclusively breastfed for at least 2 months (*n* = 17)	Culture methods, PCR of 16 s rRNA genes, qPCR using *Bifidobacterium* species-specific primers	*Bifidobacterium* species were found in all newborn/infant samples. Vaginally delivered mother-infant pairs showed monophyletic bifidobacterial strains, while none of the strains identified from C-section pairs were identified as monophyletic	[135]
Placental basal plates	Term and preterm deliveries with and without history of PROM, chorioamnionitis, group B *Streptococcus* infection, sexually transmitted infection, and/or UTIs (*n* = 195)	Histology using H&E, Gram staining, hema 3 (modification of Giemsa stain) and Brown-Hopps modification of Gram stain	27% of placentas contained intracellular bacteria in basal plate. No difference found in the incidence of bacteria in chorioamnionitis, PTB, or group B *Streptococcus* infection. There was a twofold risk increase for intracellular bacteria in very preterm birth	[38]
Meconium passed between birth and 48 h after birth	Vaginal or cesarean deliveries of preterm neonates (*n* = 52)	Ion torrent sequencing of 16S rRNA genes	67.3% of samples showed amplification of the 16S rRNA gene. Gestational age had a greater influence than mode of delivery on microbial community structure. Meconium is indicative of amniotic fluid bacterial communities	[107]
Placenta	Healthy pregnancies compared to preterm birth and history of antepartum infection (*n* = 320)	Illumina sequencing with of 16S rRNA genes and WGS metagenomics	Placentas are not sterile. Placental microbiome is associated with remote history of antenatal infection. Microbial profiles resemble oral microbiome.	[9]
Placental membranes (chorion and amnion)	Term vaginal deliveries, preterm spontaneous vaginal deliveries positive for chorioamnionitis and cesarean deliveries with intact membranes (*n* = 24)	Roche 454 FLX pyrosequencing of 16S rDNA	There was increased frequency of bacterial detection and wider spectrum of bacteria in preterm placental membranes than in term deliveries	[50]
Placental tissue, venous blood, urine, amniotic fluid	Normotensive and preeclamptic primiparous (*n* = 110)	Standard PCR and Illumina sequencing of 16S rRNA genes	12.7% of placental tissue samples from women with preeclampsia were positive by PCR, while all normotensive women were negative. Blood, urine, and amniotic fluid samples were negative except for one amniotic fluid sample colonized by *Bacillus cereus*	[150]
Posterior and side wall of vagina, inner surface of placenta, and meconium	One vaginal delivery and one cesarean delivery (*n* = 2)	Pyrosequencing of 16S rRNA genes	Placentas are not sterile. Placental and fecal samples have more diversity than vaginal samples	[151]
Meconium passed between 3 and 23 h after birth	Full-term, healthy vaginally delivered infants exclusively breastfed (*n* = 15)	FISH, standard PCR	Bacteria were detected in 66% (10 of 15) of meconium samples using FISH and 7% (1 of 10) by PCR. A higher percentage of sterile samples is observed in samples with lower MIC	[14]
Meconium, maternal and infant feces, colostrum, placenta, amniotic fluid	Full-term mother-infant pairs submitted to elective C-section (*n* = 15)	Cultures, 16S rRNA pyrosequencing, qPCR, DGGE	There were 41 bacterial phylotypes shared between meconium, amniotic fluid, and placenta. Bacterial communities of meconium and colostrum share a common maternal source; colostrum does not directly contribute to the meconium microbiota	[10]
Meconium	Vaginally or cesarean-delivered healthy full-term Japanese infants (*n* = 151)	RT-qPCR for selected species	Bacteria were detected in 95% of meconiums. The infant microbiota is strongly influenced by delivery mode. However, these differences are not noticeable at meconium stage, but become prominent at a later stage	[92]

*DGGE* denaturing gradient gel electrophoresis, *DNA* deoxyribonucleic acid, *FISH* fluorescence in situ hybridization, *PCR* polymerase chain reaction, *PROM* premature rupture of membranes, *PPROM* preterm premature rupture of membranes, *qPCR* quantitative polymerase chain reaction, *rDNA* ribosomal deoxyribonucleic acid, *rRNA* ribosomal ribonucleic acid, *UTIs* urinary tract infections, *RT-qPCR* reverse-transcription quantitative polymerase chain reaction

Seminal work by Harris and Brown [15] significantly shaped the concept of a sterile amniotic cavity by investigating the presence of cultivable bacteria in the amniotic fluid of women undergoing C-sections. All women in labor for less than 6 h (*n* = 28) tested negative for bacteria, while positive cultures were obtained from those in labor for more than 6 h (*n* = 22). Subsequent culture-based studies of amniotic fluid confirmed sterility during a healthy pregnancy [16, 17]. Specifically, bacterial culture of amniotic fluid samples obtained by abdominal puncture (*n* = 44) or by transcervical aspiration (*n* = 8) showed no growth in 96 and 50% of the cases, respectively [16]. Because fetal infection was absent, and because the positive samples were monocultures of *Staphylococcus albus*, *Streptococcus*, or yeast, the author concluded that any colonies detected resulted from contamination during collection and that his results upheld the notion of sterile amniotic fluid. Complementary to this finding, an independent investigation found an association between the presence of *Mycoplasma hominis* in the amniotic fluid and the incidence of spontaneous abortions, thereby reinforcing the notion that the presence of bacteria in the amniotic fluid should be considered an infection [17]. More recent culture-based studies reported over 90% of amniotic fluid samples tested to be sterile [18–20]. The occasional presence of a bacteria was interpreted to be due to subclinical (without maternal or fetal morbidity) [18, 19] or clinical infections [20], the latter supported by the fact that all positive cases presented symptoms of post-partum infection and pre-labor rupture of membranes [20]. Subsequent research has found that the amniotic fluid, meconium, and placental tissue contain no detectable bacteria under healthy progression of pregnancy [21–25]. When bacteria have been detected in the fetal environment, those results were obtained under circumstances where a predisposition to infection or pregnancy complications was suspected [21–25].

Because the overwhelming majority of research consistently supported the sterile womb paradigm in healthy pregnancies, later investigations into the microbiology of amniotic fluid were mostly limited to cases of pregnancy complications. These studies included cases of preterm labor (where 15% of samples were positive, *n* = 166) [26], preeclampsia (9.6% positive samples, *n* = 62) [27], small-for-gestational-age pregnancies (6% positive samples, *n* = 52) [28], preterm pre-labor membrane rupture (50% positive samples, *n* = 204) [29], and neonatal sepsis (57% positive samples, *n* = 36) [30]. Despite complications, 68% of the samples still tested negative for bacteria (as measured by cultures, polymerase chain reaction (PCR), sequencing technologies, or a combination of these methods) ([22–26], as reviewed by DiGiulio [31]).

Because the placenta is generally considered a barrier to protect the fetus from microbial pathogens that invade the blood stream of the mother [32, 33], studies directly aimed at the determination of a “placental microbiome” in healthy pregnancies are scarce. Instead, microbial studies were, for the most part, focused on complications of pregnancy or the birth process, such as spontaneous abortions (21 and 24% positive aerobic and anaerobic cultures, respectively; *n* = 47) [34], or suspected or confirmed cases of infant infection (33% positive cases, *n* = 33 and 32% positive cases, *n* = 72, as reported by [35, 36], respectively). However, even during these complications, the placentas were often found to not contain viable bacteria. In particular, Aquino and colleagues reported positive bacterial cultures (in the subchorionic fibrin of placentas) for only 11 out of 33 placentas (33%) from pregnancies where there was a suspected underlying infection and for only one of 46 (2%) healthy controls [35]. The authors concluded that most placentas are sterile, and if bacteria are present, they might originate through contamination during expulsion. Although these studies were not aimed at the determination of a “placental microbiome” in healthy pregnancies per se, their findings do reinforce the idea that bacteria are not present in the healthy fetal environment.

## In utero colonization hypothesis: the womb is not sterile and microbial colonization of the infant begins prior to birth

Most of the evidence supporting the sterile womb paradigm was generated with traditional microscopy or culture-based techniques, which are today considered deficient for assessing a microbiome. Researchers have therefore turned to molecular approaches, such as next-generation sequencing, and recent studies have produced evidence (summarized in Table 1) that challenges the sterile womb paradigm. Reports employing these techniques propose that bacteria are not only present within the fetal environment in healthy full-term pregnancies [8, 9, 37, 38], but that they also constitute a placental microbiome that jump-starts the colonization of the fetus [8–10] as part of its normal developmental process [39]. Additionally, it has been suggested that the uterus contains its own microbiome that can contribute to fetal colonization, as the placenta develops from both fetal trophoblasts and maternal decidua (the inner lining of the uterus) [40, 41].

After the early studies on the meconium discussed above, microbiological research on the meconium ceased for a period of over 30 years (reviewed by [3]) until Jimenez and colleagues reported 100% (*n* = 21) of meconiums to be positive for bacteria by culture techniques [8]. The development of molecular techniques and high-throughput sequencing spurred additional research on the microbiology of the meconium. Virtually all of these studies reported that over 90% of samples tested were positive for the presence of bacteria (Table 1), thereby seeding the idea that the placenta and the environment in which the fetus develops are not sterile.

As stated before, there are no historical studies known to us that were performed with the sole purpose of directly assessing the microbiology of the amniotic fluid from healthy pregnancies delivered at term. However, research that studied associations between microbial infection/invasion and pregnancy outcomes have occasionally included samples from uncomplicated pregnancies. Several independent studies showed that *Mycoplasma hominis* and *Ureaplasma urealyticum*, which are among several organisms highly correlated with preterm deliveries [42–44], have also been detected by culture methods or standard PCR in asymptomatic pregnancies that ended in healthy deliveries [45–48].

Recently, Collado and colleagues [10] aimed to specifically investigate microbial prenatal and neonatal transfer in an array of maternal and fetal/newborn samples from 15 full-term, healthy mother-infant pairs that submitted to elective C-section. Using 16S rRNA pyrosequencing, culture techniques, quantitative PCR (qPCR) and DGGE, the authors detected microbial populations in the amniotic fluid that were low in abundance, richness, and diversity that shared similarities with microbial populations found in the placenta. *Enterobacter* and *Escherichia*/*Shigella* were the most prevalent genera present in both placenta and amniotic fluid, followed by *Propionibacterium*. Similarities between the microbial populations found in the colostrum and meconium were also noted. The authors subsequently hypothesized that maternal intestinal microbes may be selectively transported to the mammary gland, the placenta, and the amniotic fluid, thereby contributing to an initial colonization of the fetal intestine in utero.

Aagaard and colleagues [9] were the first to use Illumina sequencing to comprehensively characterize the placental microbiome in over 300 subjects, including those with healthy pregnancies, preterm births, and cases with history of antepartum infection. The authors estimated that they isolated up to 0.002 mg of bacterial DNA per 1 g of placental tissue and detected a lowly abundant but “metabolically rich” microbiome that included *Fusobacterium* spp., *Neisseria lactamica*, *Neisseria polysaccharea*, *Rhodococcus erythropolis*, *Propionibacterium acne*, *Streptomyces overmitilis*, *Bacteroides* spp*.*, *Prevotella tannerae*, *Eschericia* sp. 4_1_408, and *Escherichia coli* (with the latter being the most abundant). This microbiome clustered with the mothers’ oral microbiota via Bray-Curtis analysis, was associated with a “remote history of antenatal infection”, and was enriched in spontaneous preterm births. These findings prompted the authors to propose that bacteria translocate by hematogenous spread from the mother’s oral cavity into the placenta and colonize the fetus in utero (Fig. 1b). In a subsequent analysis of the same samples, Aagaard and colleagues additionally concluded that the preterm placental microbiome and its metabolic profile vary with gestational weight gain [49].

In addition to sequencing and PCR, two types of microscopy-based methods have also been applied to detect bacteria in placental tissues: FISH and classic histology. Using these methods, two independent research groups [37, 38] showed that placentas from shorter gestational age deliveries were more likely to harbor intracellular bacteria compared to full-term placentas. Doyle and colleagues [50] complemented this finding by using Roche 454 FLX sequencing when they reported an increased frequency and a wider spectrum of bacteria present in preterm relative to full-term placentas.

Together, the studies above challenge the sterile womb paradigm in that they provide evidence for bacteria in the healthy in utero environment. Proponents of a fetal microbiome suggest several routes of bacterial access to the placenta, including ascension from the lower genital tract, entry through the mother’s bloodstream, or active transport of microbes by immune cells from the gut or oral cavity (Fig. 1b). It has also been proposed that the fetal-placental environment has evolved to facilitate the establishment of a diverse microbiome that further plays a role in the acquisition and assembly of the infant’s gut microbiome through in utero transmission of microbes [4, 39, 51].

## Pondering the two hypotheses

Although the sterile womb paradigm was generally agreed upon until around 10 years ago, the alternative hypothesis is experiencing a renaissance. As there is currently no clear consensus, we next evaluated the available evidence in support of each model. Several aspects must be taken into consideration; these include the anatomical, immunological, and physiological features of the placenta, the immunological status of the fetus, the limitations of the research methods used to study microbial populations, the microbiome during the very first days of life, and the evidence provided by the generation of germ-free animals (including humans).

- A. Anatomical, physical, and immunological considerations

The two main functions of the placenta are nourishment of the fetus and its protection from microbial pathogens. Accordingly, the placenta has several anatomical, physiological, and immunological features that prevent bacterial colonization.

- (i) Anatomical and physiological barriers

**Fig. 2:**
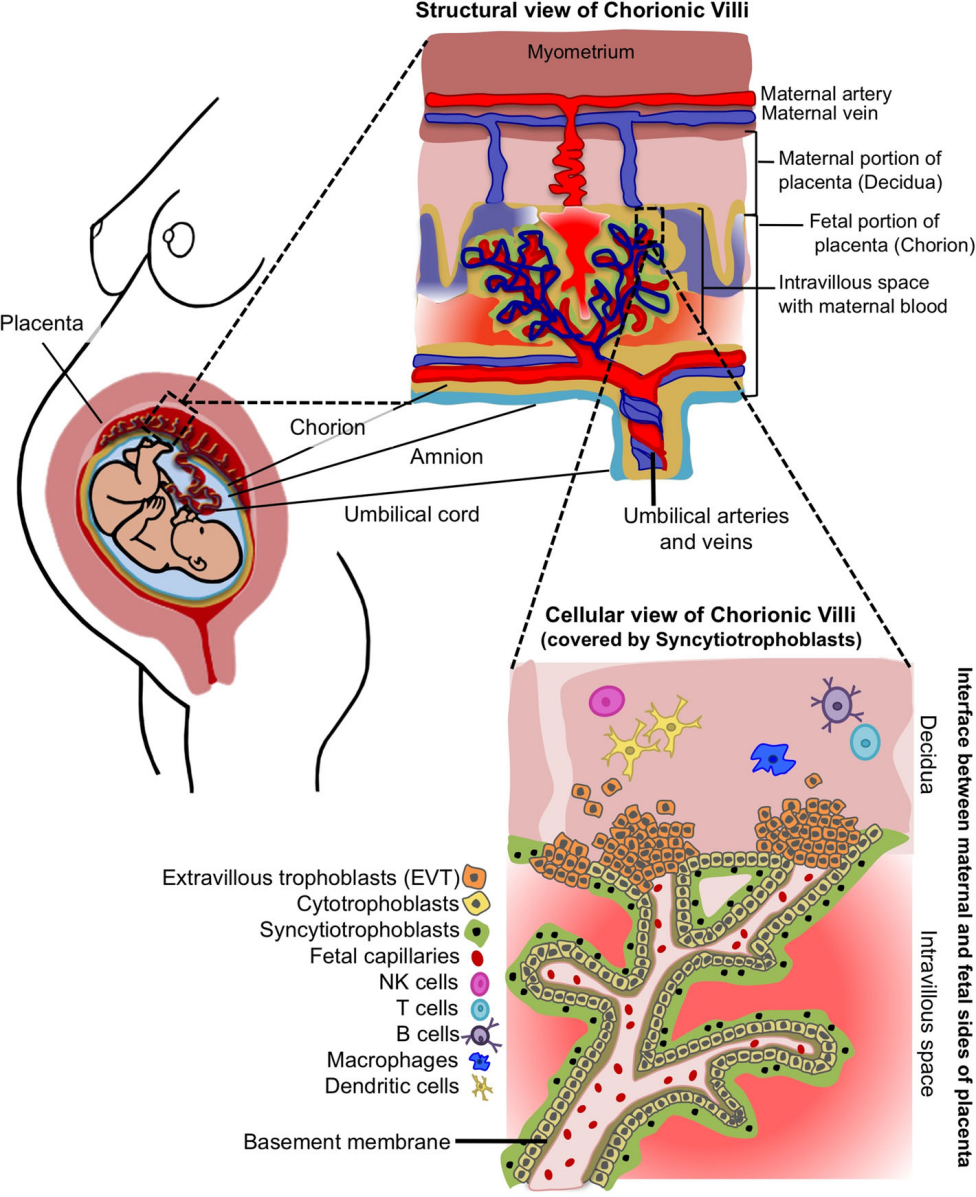
Schematic representation of the anatomical, physiological, and immunological placental barriers designed to limit microbial invasion. Three main types of cells on the fetal side of the placenta prevent access of bacterial invaders to the fetal circulation: the syncytiotrophoblast, the cytotrophoblasts, and the extravillous trophoblasts (EVT). The basement membrane also serves as a physical barrier that averts bacterial invasion. Additionally, maternal immune cells and immunoglobulins (not depicted) are near the EVTs to aid in the defense against microbial insults

The materno-fetal barrier contains two anatomically distinct elements, the chorioallantoic placenta and the chorioamnion. This barrier is formed at the placental level by the villous syncytiotrophoblast, a layer of specialized epithelial cells differentiated from underlying mononuclear cytotrophoblasts (Fig. 2). The syncytiotrophoblast actively invades the uterine wall and eventually forms the outermost fetal constituent of the placenta and the placental villi. This important epithelial layer also forms an interface between maternal blood and embryonic extracellular fluid to mediate oxygen and nutrient transfer between maternal capillaries and the fetus’ environment. Additionally, the syncytiotrophoblast acts as a continuous cell without intercellular barriers, disallowing maternal or bacterial cells to squeeze through intercellular junctions and into the fetal bloodstream. This provides a critical first level of structural protection against invasion of maternal cells carrying foreign antigens and bacterial pathogens [52–55].

Additionally, a basement membrane separates the syncytiotrophoblast from connective tissue containing fetal capillaries (Fig. 2). This placental membrane constitutes a second physical obstacle that potential microbial invaders must overcome to infect the developing fetus [33]. A third level of protection is provided by extravillous trophoblasts (EVTs). EVTs invade the decidua, functioning to anchor the placenta into the uterine wall [56] (Fig. 2). Besides being co-localized with natural killer cells, macrophages, and leukocytes, EVTs also provide innate defense mechanisms [57] and possess bactericidal properties [53, 55]. Importantly, EVTs also send tolerogenic signals to maternal leukocytes to prevent immune-mediated damage to the placenta [57].

Together, the syncytiotrophoblast, the EVT, and the basement membrane constitute the physical barrier that averts the passage of bacteria into the amniotic sac and, consequently, the fetus. Only bona fide bacterial pathogens (for example, *Listeria monocytogenes*, *Brucella abortus*, and *Toxiplasma gondii*) possess the factors necessary for successful invasion of these barriers, subversion of the immune response, and establishment in the placenta as *viable* organisms. For example, *L. monocytogenes* uses specific virulence structures such as internalins (InlA and InlB), the hemolysin listeriolysin O, and the actin assembly-inducing protein ActA to cross the intestinal, placental, and blood-brain barriers [58]. The requirement for these structures to successfully invade mammalian cells has been demonstrated by introducing them into commensal bacteria using plasmid vectors [59]. Together, these findings indicate that only pathogens and not commensals are capable of bypassing the materno-fetal anatomical barriers and establishing in the fetal environment.

- (ii) Immunological barriers

Numerous immune cells and effector molecules are present in the placenta to ensure protection against bacterial invaders. For example, toll-like receptors (TLR) 1 through 10, which are important in recognizing molecular patterns and facilitating immune responses, are present in the human placenta [60, 61], and their expression is regulated both spatially and temporally depending on gestation period [62]. Additionally, the female reproductive tract constitutively expresses antimicrobial peptides (AMP). These AMPs serve as crucial immune effectors for the placenta and fetal membranes during pregnancy by providing a chemical barrier to ascending infections [63]. The concentrations of some AMPs are increased during late gestation, while others are released directly into amniotic fluid and the fetal compartment during parturition to help defend the neonate against infection [64–66].

Other important immune effectors present in the placenta include immunoglobulins (e.g., IgG, IgA, and IgM), which play multiple important roles in regulating the course of a normal pregnancy [67, 68]. In the maternal part of the placenta, immunoglobulins protect the mother against paternal antigens present in the fetus, while in the fetal part, immunoglobulins protect the fetus against macromolecules and infectious agents originating from the mother [67]. Interestingly, most placental IgG are bound to both the trophoblastic basement membrane and the surfaces of the syncytiotrophoblast [69]. In contrast, IgM is located in the placental villous structures [70]. In particular, all of these immunoglobulins can be found as components of the outer layers of the placenta, and this location is likely a key in protecting against bacteria trying to gain access. Indeed, the presence of AMPs in the chorionic and amniotic membranes and immunoglobulins in the placenta could explain why researches have not been able to find viable bacteria in placentas from healthy, full-term deliveries. Rather than live bacteria, what may be present in placental tissues is simply bacterial products created by the antimicrobial actions of AMPs and immunoglobulins.

Altogether, the placental epithelium possesses a series of anatomical, physiological, and immunological features to prevent and combat microbial threat. Many other epithelial sites that harbor resident microbiomes also have these features. However, when entertaining the idea of a microbiome associated with the human fetus, one should consider that several immune system components needed to facilitate an “incident-free” prenatal interrelationship with the microbiome are not yet in place or mature in the fetus. Significant differences between the neonatal and adult immune system include a reduction in serum complement activity, decreased ability to produce antibodies against bacterial polysaccharide antigens, and increased numbers of naïve T cells and antigen-presenting cells with a correspondingly naïve functional program [71, 72]. Apart from a limited subset of AMPs that are expressed in distinct fetal compartments [66], fetuses do not have the immunity needed to successfully overcome bacterial invasion. Additionally, studies documenting the limited immune functions of very premature newborns indicate that the complex immune system necessary for the development of immunological tolerance to a microbiome would not be present in a fetus [72, 73, 74]. Finally, intestinal permeability is higher during the first 2 days of life for preterm infants as compared to healthy term infants [75], suggesting that the fetal gut would permit bacterial translocation and promote encounters with an underdeveloped immune system. Although there may be some evidence, albeit inconsistent, for the presence of bacterial DNA in placental and amniotic fluid samples of healthy pregnancies, it is not at all clear how an immunologically immature fetus would successfully control viable bacteria to prevent infections (and mortality) and develop normally.

- B. Methodological considerations

Most of the studies that established the sterile womb paradigm are based on microbial culture, which fails to detect viable but non-cultivable microbes. DNA-based PCR and sequencing methods overcome this limitation, and it is possible that bacteria detectable by these methods in the fetal-placental environment and meconium do represent viable, metabolically active organisms that are non-cultivable. However, one must also consider that these molecular methods have inherent limitations. First, even if bacterial DNA is detectable, the organisms could be dead. This consideration is especially relevant for the placenta, as an important role for this tissue is the removal of microbes and their components that might be present in the blood [76]. Additionally, for research to successfully challenge the sterile womb paradigm, a demonstration of microbial viability is essential, as sites can be sterile even while containing bacterial DNA. Very few groups have demonstrated viable microorganisms in the fetal environment despite these studies employing culture methods that readily grow bacteria from other parts of the body [77–83]. In the case of Satokari and colleagues [84], the authors could not culture bacteria detected by molecular methods (*Bifidobacterium* and *Lactobacillus*) even though these are readily cultivable organisms. Although the authors attribute this result to freezing the samples prior to processing, they also ponder the possibility that *only bacterial products including DNA*, *rather than living bacteria*, *are present in the placenta*. In fact, freezing samples before processing has been performed in many culture-based studies of microbiomes, and although it reduces bacterial counts, it normally does not prevent cultivation. In the case of Collado and colleagues [10], identification of bacteria cultured from the placenta and amniotic fluid of newborns delivered by C-section was limited to multiple isolates of *Propionibacterium* and *Staphylococcus* species, and one isolate each of *Streptomyces* and *Lachnospiraceae. Propionibacterium* and *Staphylococcus* species are ubiquitous normal skin commensals and could therefore originate from contamination (see below). Importantly, the authors reported that *Enterobacter* and *Escherichia*/*Shigella* were the most abundant genera detected in placenta and amniotic fluid samples. However, they were not able to recover these organisms by culture methods despite the relative ease in cultivating these bacterial groups. Taken together, these findings and other current data do not support the existence of live bacteria in the placenta.

**Fig. 3:**
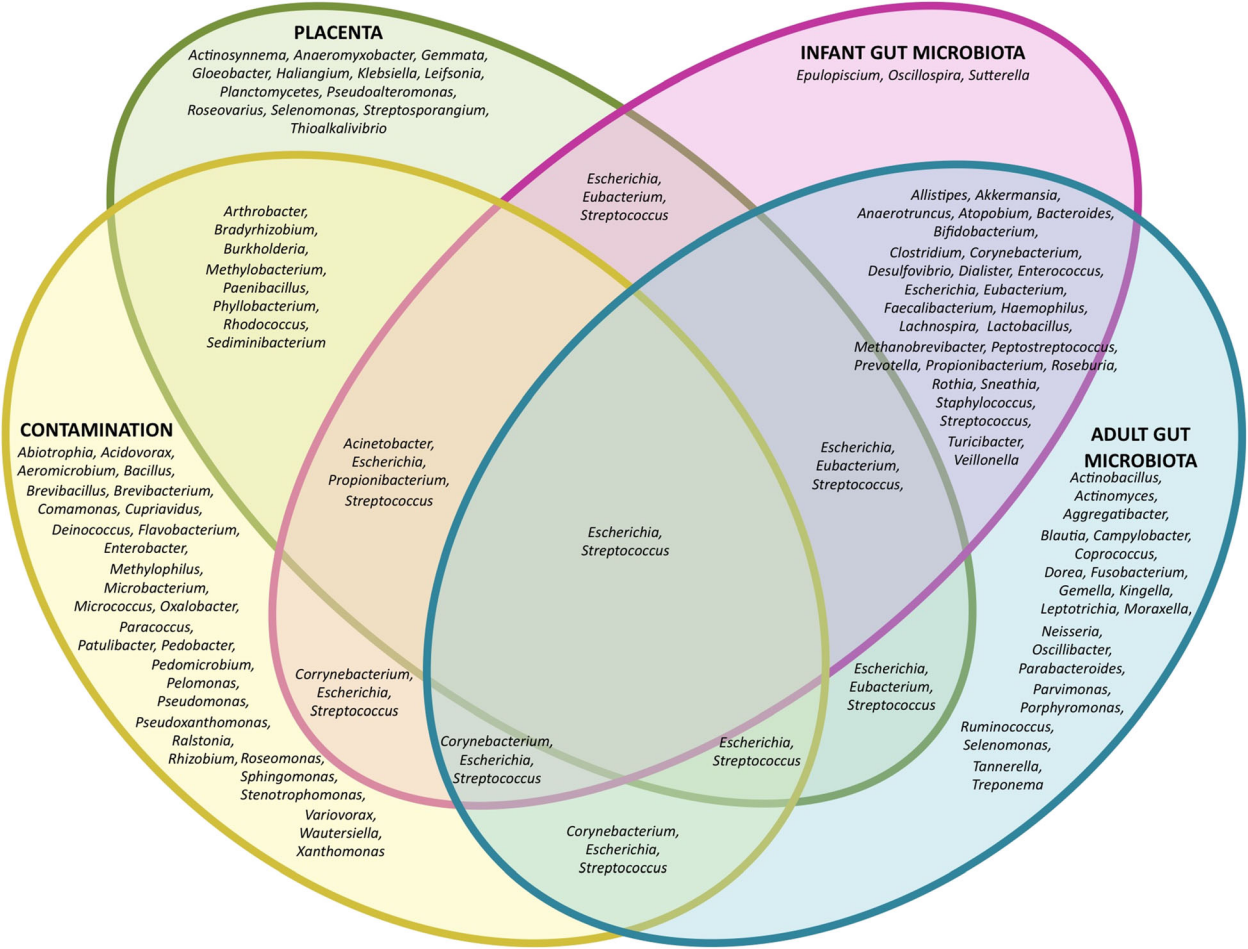
Venn diagram of bacterial genera hypothesized to contribute to the infant gut microbiome. Aagaard and colleagues [9] hypothesized that bacteria translocate from the mother’s oral cavity into the placenta, contributing to in utero colonization of the fetal gut. They further suggest that placentas contain low abundance communities of commensal bacteria. However, 36% of the bacterial genera found by Aagaard and colleagues [9] also appear on the list of contaminants found in reagents by several independent research groups as reported by Salter and colleagues [90]. Not all genera were included for each individual microbiome due to space constraints. Genera found in the infant gut [2, 101, 102, 105, 148] include taxa described in both vaginally and cesarean section-delivered babies [101, 105] and show a substantial overlap with genera found in the adult gut microbiome [145–147], but little overlap with taxa found in the placenta [9, 91] or as contaminants [85–91]

An even more important methodological consideration is that DNA-based assessments of low microbial biomass samples (such as the placenta, amniotic fluid, and meconium) are extremely prone to confounding findings from contaminant DNA. In fact, studies have demonstrated that sequence-based analyses of samples with low DNA levels are not reliable because reagents, consumables, and components of DNA extraction kits contain bacterial DNA [85–91]. Work by Salter and colleagues [90] has systematically demonstrated that the lower the amount of bacterial DNA in a sample, the higher the proportion of sequences that can be attributed to contamination. The authors provided a list of bacterial taxa commonly present in reagents and consumables that are detected in negative controls (Fig. 3). Interestingly, around 36% of the total species reported by Aagaard and colleagues [9] as “the placenta microbiome” overlap with taxa on this list. Although some researchers do report the use of controls, such as sequencing of non-template extractions [9], even these have been criticized as not sufficient [76], and most studies on the microbiota of the fetal-placental environment do not report the use of any controls [10, 38, 49, 50, 84, 92]. Clearly, lack of appropriate controls leaves the findings on fetal microbiomes extremely doubtful. This notion was recently reinforced by Lauder and colleagues [91] who systematically compared sequencing data obtained from placental samples with those from different contamination controls including sterile swabs, air swabs (swabs exposed to the air of the clinical laboratory), and extraction blanks from two different DNA isolation kits (blank tubes as a source of possible extraction/reagent contaminants). This study revealed that placenta samples contained exactly the same marginal amounts of bacterial DNA as the extraction blanks, and that the bacterial communities detected clustered closely with the contaminant community of the respective DNA isolation kit. Most importantly, no bacterial lineages were identified as unique to or present at greater abundances in placental samples when compared to contamination controls.

Apart from preventing the contamination of DNA, avoiding sample contamination per se is also a significant challenge when studying low-abundance and low-diversity bacterial communities. Samples for the study of the in utero environment are collected within a clinical setting (hospital, emergency room, delivery or operating room), making it difficult, if not impossible, to avoid sample contamination during collection and processing. In addition, the cleanliness of the tissue processing environment is particularly important in laboratories where bacterial cultures are also routinely handled. Accordingly, processing and storage time also seem to influence results. For example, Jimenez and colleagues [8] reported that their samples clustered by time of processing, as half of them were processed at the time of collection while the rest were processed 4 days after collection.

Furthermore, the method of infant delivery can also influence the degree of sample contamination and should be considered during study design. Vaginally delivered placentas are exposed to vaginal microbes during expulsion, and findings, in our opinion, can therefore not be used to argue for in utero colonization, nor should they be compared to tissues extracted via C-section. As an example, Jones and colleagues evaluated fetal and placental tissues from 74 preterm and full-term C-section and vaginal deliveries [93]. They found 30 and 43% of these tissues to be positive for bacterial DNA using qPCR. However, once the authors stratified the tissues by delivery mode, none of the full-term C-section placentas were found to be positive.

Overall, the molecular techniques used to study the fetal microbiome have inherent limitations due to their susceptibility to false positives because of contamination. In this respect, it is important to consider the limit of DNA that can be reliably detected by these methods. Even in studies that supported the presence of a placental microbiome, DNA concentrations were acknowledged to be very low [9, 10]. Therefore, only techniques capable of detecting less than 100 bacterial cells per gram of sample are likely to provide reliable results. However, even PCR methods, despite the fact that they can (in theory) detect one single DNA template, frequently have detection limits of 10^4^ to 10^6^ cells per gram when applied to samples with complex matrices [94, 95]. Although detection limits of high-throughput sequencing technologies in low-biomass samples have not been established, it is likely that they are not sufficient to reliably detect the low amounts of DNA in these samples (e.g., in the presence of contaminating DNA). Culture methods do possess the required detection limit, but as described above, most studies did not result in positive results. We therefore conclude that the in utero colonization hypothesis rests on studies that used molecular approaches with an insufficient detection limit to study “low-biomass” microbial populations and further lacked appropriate controls for contamination while failing to provide evidence of bacterial viability.

- C. Interpreting results from the study of the very early neonate’s microbiome

The repeated detection of microbes in meconium is frequently offered as evidence in support of the in utero colonization hypothesis. However, it should be recognized that only a small subset of meconiums contains detectable microbes [3, 12–14, 96]. Even if microbes are detected, bacteria in the first stool of the newborn could be the result of postnatal colonization, especially if the meconium is expelled long after delivery. Experiments with germ-free mice have shown that bacterial colonization is rapid, with bacterial species detectable 8 h after the initial exposure of the mice to conventional housing, and bacterial counts becoming equivalent to those of conventional mice after 24 h [97]. If the “germ-free human” supports microbial growth similarly to that of a germ-free mouse, then a rapid colonization process would also be expected to occur in the neonatal gut. Hansen and colleagues [14] argued that there is a “meconium colonisation interval” that provides sufficient opportunity for the microbes to multiply between rupture of the membranes during birth and the time when the meconium is delivered and processed. In support of this idea, microbial colonization of the meconium has repeatedly been shown to increase with time of passage [12, 13, 14], indicating that colonization occurs ex utero. Accordingly, studies that do not categorize and differentiate this period of time in their analyses report a higher number of positive cases [92, 98, 99].

In addition, the composition of the pioneer infant microbiome supports the sterile womb paradigm. If sterile in utero, initial inoculation of microbes is contingent upon the process of childbirth and subsequent environmental exposure (Fig. 1A) with the first major microbial exposure for a vaginally born infant occurring in the birth canal. This step is bypassed during C-sections, and delivery mode (vaginal delivery versus C-section) would therefore heavily influence the microbial composition [100–106]. In contrast, if a subset of the early microbiome were acquired in utero, then bacterial populations should be present in the infant gut that overlap with those found in the placenta/meconium, and their presence should be independent of delivery method. Some studies report that the meconium contains bacteria similar to those found in amniotic fluid [10], and authors have proposed that fetal gut colonization could occur through ingestion of amniotic fluid that contains bacteria [8, 98, 107]. However, the vast majority of the literature demonstrates that the pioneer gut microbiome is heavily influenced by birth method and later dominated by species that are characteristic gut microbes, while microbes detected in the fetal environment are absent (Fig. 3). Several studies have reported significant differences in the diversity and composition between vaginally versus C-section-delivered infants [101, 105, 106, 108], with vaginally born infants harboring an early microbiome that resemble that of the vagina, while the microbiomes of C-section infants reflect those of the human skin [101, 109]. Dominguez-Bello and colleagues showed that the bacterial communities of vaginally delivered newborns were dominated by *Lactobacillus*, *Prevotella*, or *Sneathia* species—all of which were also found in the mother’s vagina [101]. In contrast, the gut microbiota of infants delivered by C-section was dominated by the skin commensals *Staphylococcus*, *Corynebacterium*, and *Propionibacterium* [101]. Bäckhed and colleagues also reported that the gut microbiomes of infants born via C-section were dominated by skin and oral microbes as well as bacteria from the surrounding environment, while gut microbiomes of vaginally delivered infants were enriched in classical gut microbes (*Bacteroides*, *Bifidobacterium*, *Parabacteroides*, and *Escherichia*/*Shigella*) [105]. The authors further established that 72% of the early colonizers of vaginally delivered neonates could be traced back to the gut microbiota of their own mother, while this number was only 41% for C-section infants. Together, these studies convincingly show that delivery method strongly affects microbiome composition in neonates, while delivery method-independent microbial taxa originating from the placenta/amniotic fluid have not been reported. These findings support the concept of a sterile infant gut that is colonized by microbes acquired during and after birth, dependent on the environmental exposure.

- D. Considerations from the derivation of germ-free mammals

The strongest evidence against the existence of microbiomes in the fetal environment stems from the science of gnotobiology. Gnotobiology is the study of animals raised and maintained in an environment in which all microorganisms are either defined or excluded [110]. The science of gnotobiology is founded on our ability to derive germ-free animals via C-sections and subsequently raise the offspring in a sterile environment.

**Fig. 4:**
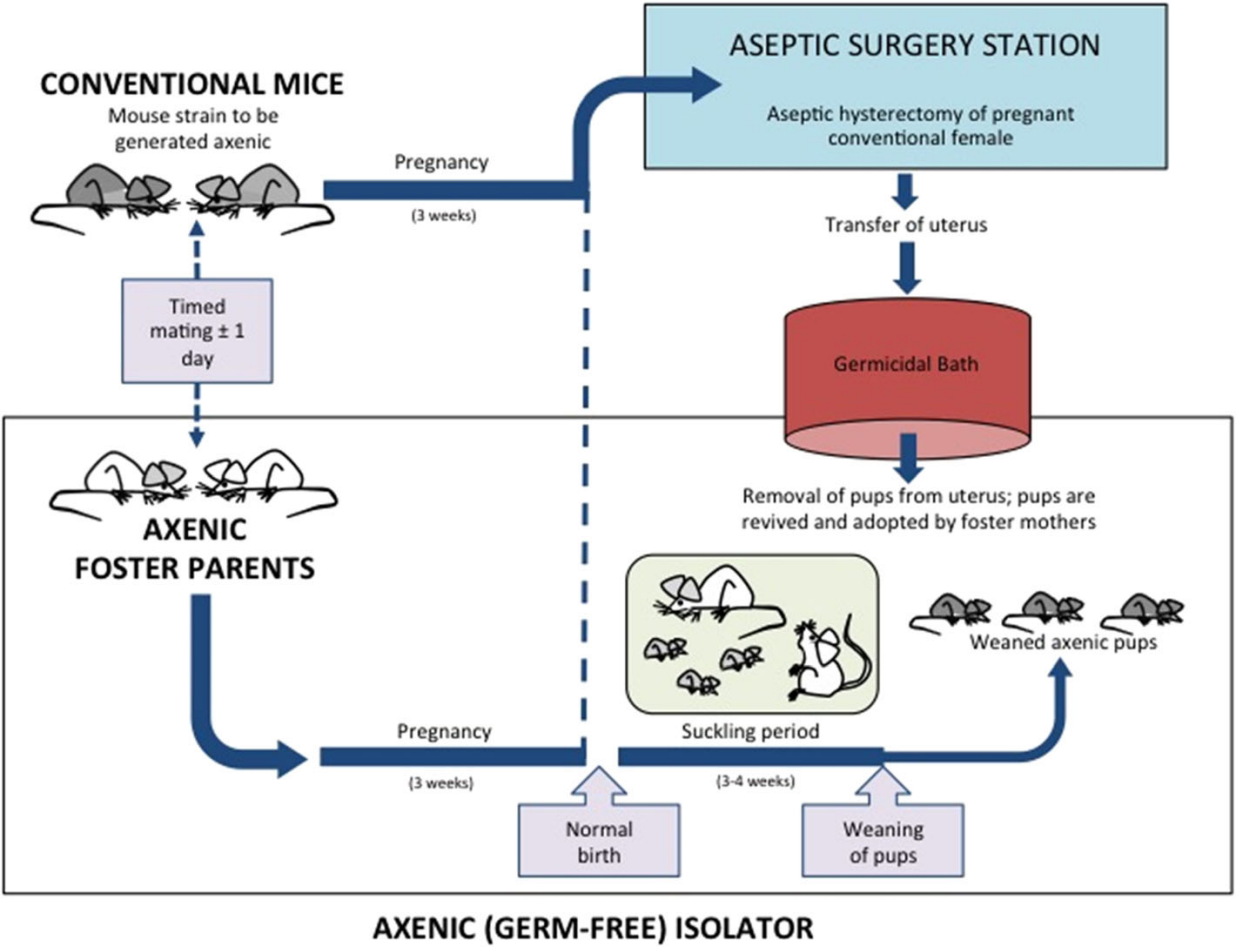
Schematic representation of the generation of axenic rodents by aseptic hysterectomy. In rodents, germ-free offspring are derived by aseptic hysterectomy. Germ-free foster mothers housed in a sterile isolator are time-mated to become pregnant in synchrony with holoxenic (conventional) females. Breeding pairs are mated on such a schedule that the aseptic hysterectomy of the donor mother can be performed a few hours before her scheduled pupping and a few hours after the foster mother gives birth. To perform the hysterectomy, donor females are euthanized, and the uterus is harvested and clamped, aseptically introduced into a germicidal bath, and then transferred into the sterile isolator where the foster mothers reside. The pups are then revived and placed under the care of the foster mother [123–125]. If there are no germ-free foster mothers available, then pups are hand-raised using sterile formula. Figure adapted from Hedrich and Hardy [125]

The first axenic animals were reported as early as the end of the nineteenth century [111], but it took until the 1940s to consistently derive axenic rodents successfully and maintain them for successive generations [112–114]. The first germ-free progenitors were generated by the labor-intensive process of C-section and hand-feeding with a sterilized artificial diet inside an aseptic isolator until maturity, after which a breeding colony was established [114]. Since then, a wide variety of animals have been successfully derived germ-free over the past 70 years, including mice, rats, guinea pigs, rabbits, dogs, cats, pigs, lambs, calves, goats, baboons, chimpanzees, and marmosets [115–122], demonstrating that the ability to derive germ-free animals is not unique to only rodent species. Currently, both commercial companies and university animal facilities routinely offer derivation as a service to the research community. Germ-free offspring can be generated from non-germ-free stock by embryo transfer and aseptic hysterectomy (Fig. 4), and aseptic hysterotomy. To perform the hysterectomy in mice, donor females are euthanized when parturition is imminent, and the intact pregnant uterus is aseptically harvested, clamped, introduced into a germicidal bath, and finally transferred into a sterile isolator. After removal from the uterus, the pups are warmed, dried to stimulate their breathing, and then placed under the care of an axenic foster mother [123–125]. Hysterotomy is usually performed to generate large axenic animals with the intent of maintaining the breeding potential of the female. In this scenario, a sterile canopy with gloves is attached to the mother’s abdomen prior to the surgery. Using sterile gloves, the surgeon makes an incision in the uterus and removes the placenta and amniotic sac, which are then transferred into a second sterile isolator so the fetus(es) can be revived in an aseptic environment [116, 119].

During hysterotomies and hysterectomies, either the intact pregnant uterus or its entire contents (including the placenta, the amniotic sac, and the fetus), respectively, are removed and transferred to generate germ-free animals. The success of these procedures provides clear evidence against the existence of a microbiome in the placenta and fetus. If microbes were present, even at low abundance, they would colonize the offspring and rapidly grow to detectable levels. This phenomenon can be observed during accidental contaminations that occur (very much to the dismay of the researchers) in germ-free animal facilities. The derivation process may involve the treatment of the non-germ-free donor female with antibiotics to reduce her microbial load prior to the hysterectomy [125]. However, oral administration of anti-microbial agents directly to the offspring is not applied, nor would this practice succeed in generating axenic offspring from animals that are already colonized. The fact that axenic animals can be derived and maintained devoid of microbes under sterile conditions provides very compelling evidence that, in most mammalian species, in utero transfer of the microbiome does not occur.

Supporters of the in utero transmission hypothesis often argue that the bacteria present in the fetal environment would potentially not colonize a germ-free host or remain undetectable after birth. However, this phenomenon is unlikely. Almost any bacterium quickly and irreversibly colonizes germ-free mice because there is no competition. Others also argue that animals are not humans, and that fetal microbiomes might be unique to humans due to physiological and anatomical differences. However, germ-free humans (although rare) have been established using protocols similar to those employed during the generation of large axenic mammals via aseptic hysterotomy [125–127]. This procedure has been applied in suspected cases of severe immune deficiency of the fetus [128]. The first germ-free human was delivered by performing a C-section inside a sterile canopy attached to the mother’s abdomen, which prevented exposure to environmental contaminants [126]. Gram stains of feces and aerobic and anaerobic cultures of swabs from both the infant and the isolator surfaces confirmed the germ-free status of the infant [126, 129, 130]. The published cases report axenic status from 6 days to 3 months after which the subjects were either removed from the axenic isolators or microbial contamination was detected [126, 127, 129, 131]. Although axenic humans are very rare for obvious reasons, the fact that they have been generated makes it extremely unlikely that humans are colonized with bacteria in utero.

## Conclusions

In 1918, Arthur Kendall summarized the contemporary knowledge on intestinal bacteriology in *The American Journal of the Medical Sciences* [132]*.* He concluded that “at birth the intestinal tract and intestinal contents are normally sterile. The first indications of bacterial contamination are recognizable several hours postpartum. The early invaders are adventitious microbes, similar in every respect to those commonly present in the infant’s environment. They gain entrance to the alimentary canal through the mouth, although the possibility of rectal infection must be borne in mind.” After having reviewed the available literature, we conclude that Kendall’s assumptions are still valid, and that there is little evidence to successfully challenge the sterile womb paradigm. The recent findings that question this premise rely mostly on (i) methodological approaches (PCR and next-generation sequencing) that do not have the detection limit necessary to study “low-density” bacterial populations, (ii) the use of methodological approaches that are extremely susceptible to contamination without the inclusion of appropriate controls, (iii) the study of samples collected in clinical settings where it is difficult to prevent contaminations, and (iv) a flawed interpretation of findings from early stool samples, which can contain microbial populations even if the fetus was sterile. Even though the bacterial species identified by molecular techniques in fetal environments are known to be readily cultivable, bacterial culture (which does provide a sufficient detection limit) is almost always negative. In our opinion, only one study published to date has used robust controls and considered low DNA levels, and the findings do not support the presence of a microbiome in the placenta [91]. Moreover, the strongest evidence against the hypothesis of a commensal placental microbiome comes from the successful generation of germ-free animals via aseptic transfer of the entire uterus (containing the placenta).

By writing this review, we aimed to contribute to the discussion of this contested topic, as we are concerned with the far-reaching implications that impact both our basic understanding of host-microbe symbiosis in humans, as well as important applied aspects such as clinical decisions and funding priorities. Transmission mode influences the mechanisms by which symbioses and mutualistic interactions evolve, as well as the extent to which environmental and lifestyle factors alter such interactions. This understanding directly informs clinical practices and recommendations, including the delivery of infants via C-sections, which have been argued by supporters of the in utero colonization hypothesis to be less detrimental than previously thought. Such discussions are not scientifically valid. Although medically necessary C-sections should not be discouraged, this procedure clearly influences establishment of the early gut microbiome [106, 133–137] and is epidemiologically linked to an increased risk for developing chronic diseases later in life [134, 137–140]. Therefore, strategies to prevent C-sections or their impact on the pioneer microbiome remain important and should be researched with the goal of preventing chronic diseases [102].

Further, it has been argued that the role of bacterial communities in the in utero environment warrants additional study [102, 141]. However, given the insufficient evidence that such communities exist, we argue that these efforts are likely futile. In our opinion, future studies (and resources) should instead focus on (i) the postnatal acquisition of the gut microbiome and its importance to health and (ii) the possible role of prenatal exposure of the fetus to microbial metabolites and compounds that originate from the maternal gut microbiota. Indeed, a recent study elegantly showed that microbial metabolites in the fetal environment can have a major impact on the development of the offspring [142]. Although the evidence does not support in utero colonization, it does however suggest an association between the presence of bacterial DNA in the placenta and preterm birth [38, 50]. Research regarding the role of this DNA would be worthwhile, but such studies must strictly control for DNA contamination during sample collection and the DNA extraction process.

Self-correction is one of science’s most fundamental principles—all findings must be subject to scrutiny and verification to determine validity [143, 144]. If a finding is incorrect, then replication will prove it as such. Unfortunately, the scientific self-correction process is slower than the transfer of information. Today, scientific findings can move freely from professional journals into the public realm (e.g., through social media), often before the scientific community has thoroughly discussed and vetted the evidence. Indeed, some of the research articles discussed in this manuscript were heavily covered in the public press. Because most members of the non-scientific community are not equipped to critique scientific findings, it is our responsibility to debate these controversial topics and facilitate the self-correction process. Failure to do so may ultimately compromise human health, damage scientific creditability, and potentially contribute to the erosion of the public’s trust in science. We hope that this review has contributed to some degree to prevent the latter.

## Abbreviations

AMP: Antimicrobial peptide
C-section: Cesarean section
DGGE: Denaturing gradient gel electrophoresis
DNA: Deoxyribonucleic acid
EVT: Extravillous trophoblast
FISH: Fluorescence in situ hybridization
PCR: Polymerase chain reaction
PPROM: Preterm premature rupture of membranes
PROM: Premature rupture of membranes
qPCR: Quantitative polymerase chain reaction
rDNA: Ribosomal deoxyribonucleic acid
rRNA: Ribosomal ribonucleic acid
TGGE: Temperature gradient gel electrophoresis
TLR: Toll-like receptor
UTIs: Urinary tract infections
